# Ipsilateral Motor Evoked Potentials as a Measure of the Reticulospinal Tract in Age-Related Strength Changes

**DOI:** 10.3389/fnagi.2021.612352

**Published:** 2021-03-03

**Authors:** Stuart Maitland, Stuart N. Baker

**Affiliations:** ^1^Translational and Clinical Research Institute, Newcastle University, Newcastle upon Tyne, United Kingdom; ^2^Biosciences Institute, Newcastle University, Newcastle upon Tyne, United Kingdom

**Keywords:** transcranial magnetic stimulation, reticulospinal tract, ipsilateral motor evoked potential, sarcopenia, grip strength

## Abstract

**Background**: The reticulospinal tract (RST) is essential for balance, posture, and strength, all functions which falter with age. We hypothesized that age-related strength reductions might relate to differential changes in corticospinal and reticulospinal connectivity.

**Methods**: We divided 83 participants (age 20–84) into age groups <50 (*n* = 29) and ≥50 (*n* = 54) years; five of which had probable sarcopenia. Transcranial Magnetic Stimulation (TMS) was applied to the left cortex, inducing motor evoked potentials (MEPs) in the biceps muscles bilaterally. Contralateral (right, cMEPs) and ipsilateral (left, iMEPs) MEPs are carried by mainly corticospinal and reticulospinal pathways respectively; the iMEP/cMEP amplitude ratio (ICAR) therefore measured the relative importance of the two descending tracts. Grip strength was measured with a dynamometer and normalized for age and sex.

**Results**: We found valid iMEPs in 74 individuals (*n* = 44 aged ≥50, *n* = 29 < 50). Younger adults had a significant negative correlation between normalized grip strength and ICAR (*r* = −0.37, *p* = 0.045); surprisingly, in older adults, the correlation was also significant, but positive (*r* = 0.43, *p* = 0.0037).

**Discussion**: Older individuals who maintain or strengthen their RST are stronger than their peers. We speculate that reduced RST connectivity could predict those at risk of age-related muscle weakness; interventions that reinforce the RST could be a candidate for treatment or prevention of sarcopenia.

## Introduction

### Sarcopenia

Sarcopenia (“poverty of flesh”) is the progressive degenerative loss of muscle mass and strength associated with aging. As part of the frailty syndrome, sarcopenia is a major obstacle to the independence, quality of life, and longevity of aging adults, who are more likely to require hospitalization and additional care (Shafiee et al., 2017; Cruz-Jentoft et al., 2018). For example, age-related muscle weakness is the leading contributing factor to falls (Rubenstein, 2006), and one in three people over the age of 65 suffer a fall, with an economic burden to the UK’s National Health Service (NHS) of £2.3 billion (NHS England, 2019).

Muscle mass has previously been the primary factor in defining sarcopenia; however, with aging, there is a disproportionate loss of muscle strength compared to mass (Metter et al., 1999). Muscle mass alone is not sensitive in finding individuals with functional limitations due to sarcopenia (Hughes et al., 2001). The European Working Group on Sarcopenia in Older People (EWGSOP) has recently updated the definition of sarcopenia to place primacy on muscle strength and functional parameters rather than muscle bulk (Cruz-Jentoft et al., 2018). This new definition is termed muscle “quality over quantity,” or dynapenia (Clark and Manini, 2008; “poverty of strength”).

Several recent reviews have commented on whether sarcopenia is primarily a neurological process rather than a muscular one (Clark and Manini, 2008; Clark and Fielding, 2012; Carson, 2018). Briefly, motor system changes with age involve cortical factors such as reduced central activation (Stevens et al., 2003), spinal factors including loss of spinal motor neurons (Henneman et al., 1965; Kawamura et al., 1977; Tomlinson and Irving, 1977), and peripheral factors which include reduced nerve conduction velocity (Metter et al., 1998; Palve and Palve, 2018), remodeling of the motor unit with de/reinnervation (Piasecki et al., 2016), and impaired neuromuscular transmission (Willadt et al., 2018).

Clinical features of sarcopenia include reduced gait speed (Abellan Van Kan et al., 2009; Peel et al., 2013), balance (Mathias et al., 1986), and difficulty rising from a chair (Jones et al., 1999; Cesari et al., 2009). Although these activities all require complex motor coordination, they all involve control of proximal and axial muscles.

### The Reticulospinal Tract (RST)

Motoneurons are innervated by descending spinal pathways. The pyramidal corticospinal tract is dominant for the volitional control of motoneurons (Wiesendanger, 1981), with significant contributions from extrapyramidal pathways including the vestibulospinal and reticulospinal tracts. It is known that corticospinal fibers are lost with aging (Terao et al., 1994), however loss of fibers in other pathways is not well characterized.

The reticulospinal tract (RST) operates bilaterally (Jankowska et al., 2003; Davidson et al., 2007), diverging in the spinal cord to innervate large groups of muscles in synergistic patterns (Peterson et al., 1975). This contrasts with the dominant contralateral corticospinal projections which innervate small groups of motoneurone pools (Shinoda et al., 1981; Buys et al., 1986), allowing fractionation of fine-grade movements (Zaaimi et al., 2018). The RST is involved in postural control (Prentice and Drew, 2001; Schepens and Drew, 2004, 2006) and muscle tone during gait (Takakusaki et al., 2016). It also contributes to motor control of upper limb muscles (Honeycutt et al., 2013; Dean and Baker, 2017). There is a presumed proximal dominance of the RST; however this has not been proven, and RST projections have even been found for the intrinsic hand muscles (Riddle et al., 2009).

Neural adaptations to strength training have been demonstrated before intramuscular changes (Moritani and DeVries, 1979; Sale, 1988). Changes in motor cortical excitability (as assessed by MEP amplitude in Kidgell et al., 2017) and cortical inhibitory networks (as assessed by short-interval intracortical inhibition and ipsilateral silent period in Nuzzo et al., 2017) occur during strength training, but not in corticospinal connectivity [as assessed by Transcranial Magnetic Stimulation (TMS) latency in Kidgell et al., 2010]. Consistent with this work in humans, our group recently showed that resistance training in two female nonhuman primates results in intracortical and reticulospinal adaptations, but no change in the corticospinal tract and motoneurone excitability (Glover and Baker, 2020). Based upon this known role of the reticulospinal tract in strength and posture, we wondered whether it might also drive centrally-based changes in sarcopenia.

### Ipsilateral Motor Evoked Potentials (iMEPs)

The study of the different descending pathways in humans requires non-invasive ways to assess their function. Transcranial Magnetic Stimulation (TMS) uses brief high-intensity magnetic fields to excite cortical neurones (Barker et al., 1985; Kobayashi and Pascual-Leone, 2003). When applied over the primary motor cortex (M1), single-pulse TMS excites the corticospinal tract and elicits motor evoked potentials (MEPs) in contralateral muscles, which are commonly used to assess corticospinal function (Edgley et al., 1997).

TMS can also elicit iMEPs, particularly when facilitated by strong background contraction (Wassermann et al., 1991, 1994), antagonistic (Tazoe and Perez, 2014), or phasic movements (Bawa et al., 2004). iMEPs have higher thresholds for activation and longer latencies than contralateral MEPs (cMEPs), with previous studies of iMEPs using either 10% above active motor threshold (Carr et al., 1994; Bawa et al., 2004) or 100% of maximum stimulator output (Wassermann et al., 1991; Tazoe and Perez, 2014). The amplitude and latency of iMEPs are modulated by rotating the head (Ziemann et al., 1999; Tazoe and Perez, 2014), consistent with prior work demonstrating that reticulospinal cells are modulated by neck proprioceptors (Pompeiano et al., 1984; Srivastava et al., 1984).

This latency and facilitation pattern is consistent with transmission *via* a brainstem relay and the reticulospinal tract, which would add additional synaptic delay. TMS delivered over M1 can activate reticulospinal cells transsynaptically *via* corticoreticular connections (Fisher et al., 2012). The reticulospinal tract originates from multiple nuclei in the ponto-medullary reticular formation and projects to the cord bilaterally (Sakai et al., 2009; Baker, 2011). This extensive divergence explains why ipsilateral muscle responses can be seen even when the stimulus activates only one cortical hemisphere and suggests that iMEPs could be used to assess reticulospinal function (Ziemann et al., 1999).

This was an exploratory study measuring ipsilateral and contralateral motor evoked potentials (iMEPs/cMEPs) as a method of assessing reticulospinal and corticospinal contributions to age-related muscle strength losses in healthy younger and older adults.

## Materials and Methods

### Subjects

Ethical approval was obtained from the Newcastle University Medical Faculty ethics committee (approval number 14189/2018). We recruited candidates from a local volunteer pool. Exclusion criteria were based upon safety criteria for TMS (Rossi et al., 2011), including implanted medical devices and any history of epilepsy or other neurological condition. The clinical investigator (SM) conducted a brief medical interview to assess for any neuromuscular conditions or medications that may impact synaptic transmission. We explained the purpose and procedure of the experiment, and all subjects signed informed consent to participate.

### Anthropometry and Sarcopenia Stratification

We stratified all participants over the age of 50 using the revised European Working Group on Sarcopenia in Older People (EWGSOP) criteria for sarcopenia (Cruz-Jentoft et al., 2018). The first step of this was to use the SARC-F 5 item questionnaire to screen for self-reported functional limitations of sarcopenia, namely strength, walking, chair rise, stair climbing, and falls (Malmstrom et al., 2016).

Handgrip was chosen to measure strength as it is a widely used clinical measure, and forms part of sarcopenia diagnostic criteria (Cruz-Jentoft et al., 2018). Participants self-reported their hand dominance. In all individuals, grip strength (F) was measured only in the participant’s self-reported dominant hand using a Jamar hydraulic dynamometer (J. A. Preston Corporation, New Jersey, NJ, USA). Following revised EWGSOP criteria (Cruz-Jentoft et al., 2018), we considered all individuals with grip strength below a set level (male <27 kg, female <16 kg) to have probable sarcopenia.

Grip strength was standardized to age and sex using the “LMS” method relative to a large UK population dataset (Dodds et al., 2014). Used initially in describing child growth centiles (Cole, 1989), LMS estimates population distributions using the age and sex-specific skew (*L*), median (*M*), and generalized coefficient of variation (*S*) to produce an individual *z-score* from measured force *F*, using the following formula:

$$z={\left(F/M\right)}^{L}-\text{1}/\left(S\times L\right)$$

Whole-body muscle mass was estimated using anthropometry *via* a Tanita BC-545n Bioimpedance Analyser (Tanita Europe, Amsterdam, The Netherlands) which utilizes the differential impedance of electrical current through the body to estimate the lean muscle mass (LMM) and the total weight of the participant. These impedance values have been calibrated on a European population group (Tanita, 2020). Muscle mass is strongly correlated with height, and so was standardized similarly to BMI (LMM/height^2^) as previously described in the literature (Kim et al., 2016).

### EMG and TMS Measurements

We recorded electrical signals using Ag/AgCl electrodes (50 mm Covidien H34 SG) attached bilaterally across the belly of both biceps brachialis. The center of each electrode was separated by 10 cm. This muscle was selected for its involvement in performing the rowing task, and because iMEPs have been reported in healthy subjects using this muscle previously (Turton et al., 1996; Ziemann et al., 1999; Bawa et al., 2004; Tazoe and Perez, 2014).

For TMS, we used a Magstim 200^2^ with a figure-of-eight coil, winding diameter 70 mm, placed on the left side of the head to produce a right-sided cMEP and left-sided iMEP. The coil was placed tangential to the scalp and at 45° to the midsagittal plane, so that induced current flowed from posterior-lateral to anterior-medial. This placement was irrespective of the subject’s handedness.

The subject wore a headband with reflective markers, with similar markers on the TMS coil, with coil position mapping *via* the Brainsight neural navigation system (Rogue Industries, Montreal, QC, Canada). The structural shape of the subject’s head was mapped using pointers. The “average MNI head model” was loaded within Brainsight, but this was not used for navigation relative to brain structures; rather, the Brainsight system merely allowed us to maintain the coil over the hot spot, defined relative to the skull. The coil was held in place manually with visual feedback to maintain coil position fixed relative to the head, despite participant movement. We then localized the motor cortical representation of the biceps by optimizing coil placement with visual feedback from the neural navigation system to produce a maximal amplitude MEP in the contralateral biceps during weak contraction.

Once we located the hot spot, we determined the active threshold as being the level of TMS output to the nearest 5% of maximum stimulator output that produced an MEP in contralateral biceps during a sustained weak contraction with amplitude greater than 0.3 mV in at least three of six stimuli. This was chosen to prevent coil overheating later in the experiment due to overuse, as previously proposed (Groppa et al., 2012).

### Rowing Task

Subjects used a seated resistance exercise training machine in a rowing configuration, which provided 12 kg of resistance per arm. The rowing machine was configured with two handles and resistance bands to ensure both arms contributed equally and separately to the rowing action, and subjects arm position was observed during the task to ensure consistent bilateral movement. All subjects rowed against the same fixed resistance level. Subjects performed a bilateral rowing movement against this resistance, with movement start cued by an auditory beep. They maintained a vertical torso position and kept hands supinated in order to activate biceps during the contraction.

**Figure 1:**
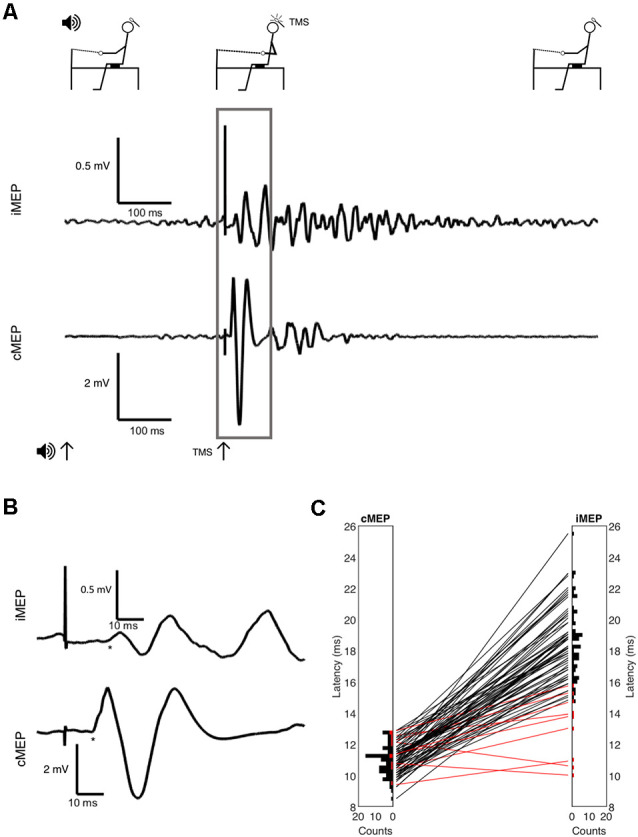
**(A)** Full experimental paradigm showing subject position, the onset of auditory cue at *t* = 0, the onset of Transcranial Magnetic Stimulation (TMS) at *t* = 300 ms, and EMG. Volitional EMG activity builds after auditory cue, with large synchronous motor evoked potential (MEP) after TMS indicated and inset in panel **(B)** with selected area and onsets marked with * symbol. **(C)** Latency histograms and differences between contralateral MEP (cMEP) and ipsilateral MEP (iMEP) latencies shown. Each line is a subject, with iMEPs deemed invalid due to low latency difference (<5 ms) in red.

Head position was maintained at 30° to the left of midline (towards the left biceps). Maximal neck turn maximizes iMEP amplitude (Tazoe and Perez, 2014), however this angle was chosen to be safe and feasible while remaining consistent with previous investigations on neck angulation on muscle tone (Aiello et al., 1988). We provided an audible, but non-startling auditory start stimulus at intervals of 5–6 s randomly chosen from a uniform distribution, to avoid anticipation (Awiszus, 2003). TMS was applied 300 ms after the auditory cue in order to occur during the subjects’ movement. A summary of the experimental protocol is shown in Figure 1A.

Twenty movements with TMS were performed at each TMS intensity (0, 5, 10, 15, 20, and 25% of maximum stimulator output above the active motor threshold) in two blocks of 10 TMS pulses, for a total of 120 stimuli per subject. To collect background activity, some movements without TMS (audio cue only) were scattered within blocks, meaning up to 14 movements could be performed per block, including exactly 10 movements with added TMS stimuli, and two to four movements without TMS.

The sequence of blocks was arranged randomly but identical between participants. In total, subjects performed 160 rowing movements, and the experiment lasted up to 16 min. Participants were observed and questioned for any self-reported fatigue during this with ample opportunity for breaks during the experiment.

EMG activity was amplified using a Digitimer D360 amplifier (Gain 300, bandpass 30 Hz – 2 kHz) and sampled at 5 KHz using a micro1401 interface [Cambridge Electronic Design (CED), Cambridge, UK] which also controlled the TMS pulse and auditory cue, programmed using Spike2 sequencing software (also CED).

### Data and Statistical Analysis

Data were exported and analyzed using Matlab R2019a. The twenty unrectified MEPs within each stimulus intensity were averaged, as were the forty background EMG traces.

To assess validity of iMEPs, a custom program presented averaged MEPs within each TMS intensity and electrode in a random order to one of the authors (SM), who determined onset latency subjectively by the point of divergence from background activity. The rater was blinded to identifying features of participant and laterality and marked onset latency with a cursor.

Matlab was used to measure peak-to-peak amplitudes in the subsequent 10 ms of MEP. We then selected the TMS intensity for each participant which elicited the highest valid iMEP amplitude for further analysis. Although cMEPs recruit to higher amplitudes with increased TMS intensity, this is not necessarily true for iMEPs, which may be affected by the ipsilateral cortical silent period (Wassermann et al., 1991; Meyer et al., 1995). Interhemispheric inhibition (IHI) increases with higher intensities (Ferbert et al., 1992), as does iMEP amplitude. Depending on which process increases faster, iMEPs could either grow or diminish with increases in stimulation strength. This means that the highest TMS intensity does not always yield the largest iMEP.

There was no minimum amplitude for an iMEP. Although this has been used previously (Bernard et al., 2011), we felt that in older adults the reduced peripheral nerve conduction velocity (Palve and Palve, 2018) leading to dispersion and lower compound muscle action potential amplitude (Taylor, 1993), would limit the validity of these measures.

After calculating the latency difference between onset of cMEP and iMEP, we then rejected data from subjects where this was less than 5 ms as being possibly due to direct activation of the contralateral cerebral hemisphere by current spread (Turton et al., 1995; Ziemann et al., 1999) rather than a true iMEP. While iMEP latencies more than 10 ms longer than the cMEP are theoretically compatible with a transcallosal pathway on the basis of latency alone (Ziemann et al., 1999), this route is unlikely. iMEPs have been detected even in patients with agenesis of the corpus callosum (Ziemann et al., 1999), and transcallosal effects are usually inhibitory, especially at higher intensities (Ferbert et al., 1992; Sohn et al., 2003). Accordingly, no upper bound was set for the latency difference.

The amplitude of iMEPs and cMEPs is affected by many factors, such as excitability of motoneuron pools (Eisen et al., 1991) and peripheral factors that could limit the compound muscle action potential such as muscle density (Yuen and Olney, 1997) and subcutaneous fat (Nordander et al., 2003). To correct measurements for this, we calculated the ipsilateral to contralateral amplitude ratio (ICAR) as previously described (Bawa et al., 2004). Greater ICAR values indicate higher reticulospinal control of muscles, whereas lower ICAR values indicate higher corticospinal control of muscles.

We calculated Pearson correlations between clinical measurements (grip strength, standardized grip strength, height, lean muscle mass) and ICAR. We also compared categorical variables between age groups with Fisher’s exact test. Continuous anthropometric measurements were tested for normality using the Anderson-Darling test (Anderson and Darling, 1952).

## Results

### Participant Characteristics

**Figure 2:**
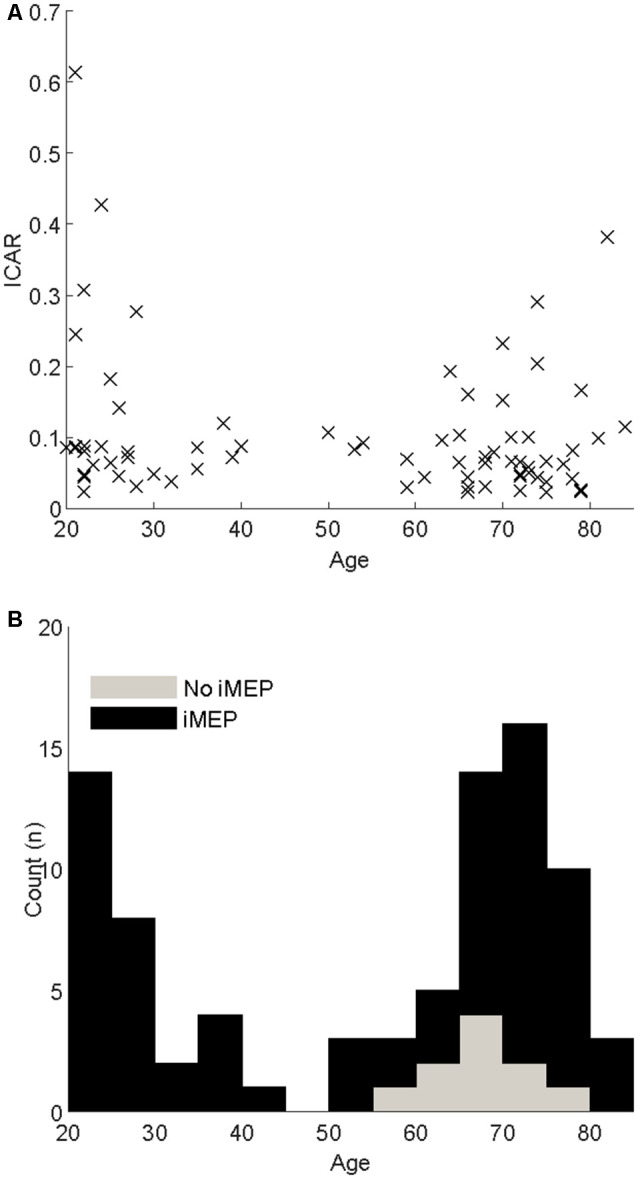
**(A)** Scatter of the Ipsilateral-Contralateral Amplitude Ratio (ICAR) changes with age. **(B)** Stacked histograms indicating the age distribution of participants. Black bars indicate participants where iMEPs were elicited; gray indicates participants without iMEPs.

There were 83 participants; descriptive characteristics are listed in Table 1. A bimodal distribution of ages was seen, with means around ages 20 and 70 (Figure 2). The experiment was well tolerated, and no participants experienced any adverse symptoms [syncope, seizure and transient hearing changes have previously been reported (Rossi et al., 2009)] or withdrew from the investigation. We elicited valid iMEPs (see “Materials and Methods” section for latency criteria) in 73 participants. As expected, raw grip strength was significantly different between age groups (*p* = 0.01); however other anthropometric measurements, including height, weight, gender and handedness were statistically similar (Table 1). Normality testing failed to reject the null hypothesis at 5% significance level.

**Table 1:** Participant characteristics.

	A			B	Age ≥ 50	
	Age< 50 (*n* = 29)	Age ≥ 50 (*n* = 54)	*p*	iMEP (*n* = 44)	No iMEP (*n* = 10)	*p*
Male	11	21		17	4
Female	18	33	1.00^F^	27	6	1.00^F^
Right-handed	27	52	0.61^F^	42	10	1.00^F^
Height (SD)	170 (9.1)	166.7 (13.4)	0.24	167.9 (9.7)	161.7 (23.8)	0.19
Weight (SD)	67.1 (15)	69.1 (23.4)	0.68	70 (23.4)	65.1 (24.2)	0.56
LMM (SD)	47.8 (8.4)	49.15 (10.5)	0.55	44.4 (17.3)	45.2 (19.5)	0.90
Grip-kg (SD)	35.2 (9.3)	29.6 (9.03)	**0.01**	29.5 (8.9)	30 (11.3)	0.89
Grip-z (SD)	−0.11 (1.04)	0.15 (1.12)	0.39	0.15 (1.11)	−0.17 (0.77)	0.39
Valid iMEP	29	44	**0.013**^F^	−	−	−
cMEP threshold (SD)	69.5 (13)	63.7 (14.1)	**0.028**	63.6 (13.9)	56.5 (13.6)	0.14

*All participants compared in *(A)*, older participants divided by ipsilateral Motor Evoked Potentials (iMEPs) response in *(B)*. All values were compared with the Student’s t-test, except categorical measures of gender and handedness indicated with F which were compared with Fisher’s exact test. Bold indicates significant values (*p* < 0.05)*.

Our participant population was representative, and z-scores of grip strength followed the normal distribution with 55 (66.2%) within the range [−1, + 1] and 79 (95.2%) within the range [−2, + 2]. The Anderson-Darling test for normality failed to reject the null hypothesis. Five participants met revised EWGSOP criteria for probable sarcopenia, based upon low grip strength.

### Ipsilateral Motor Evoked Potentials (iMEPs)

We were interested to see if the reticulospinal system was implicated in age-related muscle weakness. Aging affects several parts of the motor system, and it was necessary to delineate these differences first.

cMEP thresholds were significantly (*p* = 0.026, *t*-test) higher in the younger age group (69.5%, 95% CI 64.52–74.45, Cohen’s *d* effect size 0.51) compared to the older age group (63.7%, 95% CI 58.5–66.13). The TMS intensity required to produce maximal iMEP was 80.1% (95% CI 76.9–83.3) across ages, 86.9% (95% CI 82.5–91.2) in those aged <50, and 76.5% (95% CI 72.4–80.6) in those aged ≥ 50. This was significantly different between age groups (*t* -test *p* = 0.0016, Cohen’s *d* effect size 0.71).

We elicited iMEPs from all 29 participants in the younger age group, compared to 44 of 54 older participants. The iMEP success rate was significantly different between age groups (*p* = 0.013, Fisher’s exact test). We examined this subgroup closer to check whether iMEP non-responders differed from responders, but the two subgroups were of comparable age, gender, handedness and grip strength, with no statistically significant differences (Table 1B). While the experiment was not altered for handedness, all data were measured *post-hoc* to ensure that removal of left-handed individuals did not significantly alter results.

Example MEPs are shown in Figures 1A,B, demonstrating the longer latency and reduced amplitude of iMEPs compared to cMEPs. Mean iMEP amplitude was 0.29 mV (95% CI 0.238–0.349). In ages <50 this was 0.361 mV (95% CI 0.262–0.459) and 0.248 mV (95% CI 0.181–0.315) in those aged ≥50. This just failed to reach statistical significance (*t*-test, *p* = 0.0505).

iMEP latency followed a much wider distribution (mean 18.6 ms, SD 2.3) compared to cMEPs (mean 10.9 ms, SD 0.95; *F*-test for equality of variance, *p* < 0.001; Figure 1C). The mean iMEP latency in ages <50 was 17.8 ms (95% CI 16.8–18.7) compared to 19.1 ms (95% CI 18.4–19.8) in ages ≥50. This was significantly different (*t*-test *p* = 0.022, Cohen’s *d* effect size 0.61).

### Ipsilateral/Contralateral Amplitude Ratios

**Figure 3:**
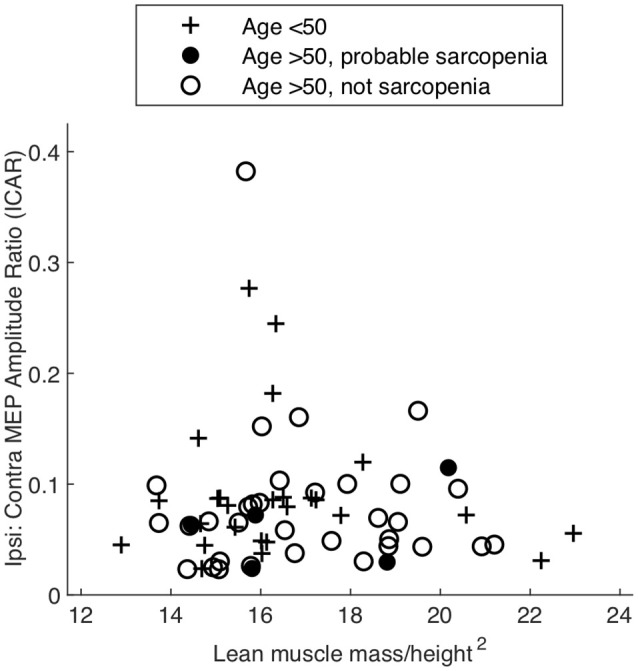
Amplitude ratio (ICAR) compared to a measure of muscularity (lean muscle/height^2^) in different age groups and sarcopenia.

ICAR values varied widely between individuals (range 0.02–0.43) but were comparable to previous reports (Wassermann et al., 1994; Bawa et al., 2004). This experiment involved a fixed-weight resistance task, and since MEP behavior is dependent on the degree of voluntary muscle activation, it was important that any differences observed were not simply a result of differing ability to complete the rowing task. ICAR values across age groups were not significantly correlated with LMM/height^2^, as shown in Figure 3 (*r* = −0.196, *p* = 0.097), and unrelated to gender (*p* = 0.833, *t* -test). ICAR was also uncorrelated with age (*r* = −0.175, *p* = 0.138), height (*r* = −0.044, *p* = 0.713) or raw uncorrected grip strength (*r* = 0.011, *p* = 0.924).

**Figure 4:**
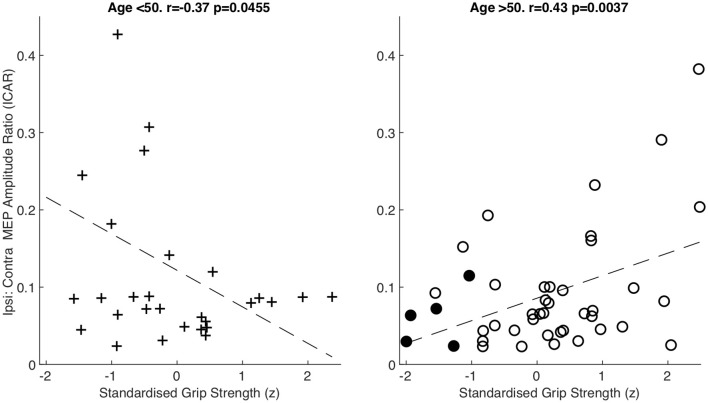
Amplitude ratio (ICAR) compared to standardized grip strength in both age groups. Sarcopenic patients are highlighted with filled circles.

After splitting data into age groups, we discovered an intriguing relationship between ICAR and strength measures. Raw grip strength showed no relationship with ICAR values in younger (*r* = −0.203, *p* = 0.292), or older (*r* = 0.145, *p* = 0.348) age groups. However, raw grip strength has a strong dependence on age and sex (Dodds et al., 2014), which could obscure a more subtle relationship with ICAR. We therefore calculated a standardized *z* score as described in “Materials and Methods” section, which removed the influence of age and sex. This *z* score measures whether an individual is stronger or weaker than would be expected, given the range of strengths seen across a population of the same age and sex. In the younger cohort, there was a significant negative correlation between standardized grip strength and ICAR (*r* = −0.37, *p* = 0.045; Figure 4). Surprisingly, in the older cohort this correlation was also significant, but of the opposite (positive) sign (*r* = 0.43, *p* = 0.0037; Figure 4). The two correlation coefficients were significantly different (*p* = 0.049) when compared using Fisher’s method (Fisher, 1921). Younger subjects who were stronger than expected for their age and sex had smaller ICAR; by contrast, stronger older subjects had greater ICAR.

## Discussion

In this study, we assessed RST function non-invasively with TMS and a bilateral rowing task to record iMEPs. For the first time in humans, we have demonstrated that strength is related to non-invasive measures of RST function, which differ between younger and older people.

### Key Findings

In younger adults, those with lower standardized grip strength had higher ICAR values. This was unexpected, given our previous work which showed that strength training enhances the RST (Glover and Baker, 2020); we might therefore have expected the stronger individuals to have larger ICAR, reflecting more prominent RST projections. However, it must be remembered that ICAR is a measure of the ratio of iMEP and cMEP amplitude. In healthy young monkeys, we previously showed that strength training led to an increase in cortical excitability and thus elevated cMEPs (Glover and Baker, 2020). A careful meta-analysis also revealed marginal evidence for increased cMEPs after strength training in humans (Kidgell et al., 2017). Particularly strong young subjects may have both enhanced cMEPs and iMEPs, leading to an unremarkable ICAR which was similar to those of more typical strength.

By recruiting a relatively large number of subjects, we were able to demonstrate significant correlations with functional measures even in the face of considerable inter-subject variability. This includes demonstrating a significantly lower active motor threshold for both cMEPs and iMEPs in older participants, in contrast to previous studies that have shown no relationship (Matsunaga et al., 1998; Wassermann, 2002; Pitcher et al., 2003). While this could be due to sulcal effacement with age (Parashos and Coffey, 1994) reducing the distance to the TMS focal point, the reduction in brain volume with age (Svennerholm et al., 1997) would be expected to counter this effect, and further investigation is needed to assess this.

As previously reported, iMEPs exhibited a significantly longer latency than the corresponding cMEP in the majority of individuals, supporting mediation by an indirect pathway involving the RST (Ziemann et al., 1999). While iMEP latency was significantly longer in older individuals, this is likely due to reduced peripheral nerve conduction velocities with age (Palve and Palve, 2018).

### Potential Pathophysiologies

One potential explanation for the high ICAR measures in weaker young subjects is that these individuals were physically weaker due to non-neural factors (e.g., nutrition or genetic factors influencing muscle physiology). As many daily activities require a fixed level of activation, these subjects may have been exposed effectively to a strength training regime merely by performing their activities of daily living. Regularly performing contractions close to maximum effort could have led to the strengthening of the RST, as we have previously reported (Glover and Baker, 2020). If so, this neural adaptation would render these subjects stronger than they would be otherwise, but still weaker than their peers. The situation is perhaps reminiscent of the correlation which we have reported previously in stroke patients, were more severely affected patients show an enhanced RST projection (Choudhury et al., 2019) which likely allows some restoration of function. If so, the negative correlation seen here in young subjects reflects compensation for weakness, rather than part of the underlying cause of weakness itself. A further possibility is that the primary weakness was caused, not only by peripheral factors but also by mild corticospinal loss. This would also lead to RST strengthening, and further, elevate the RST: CST ratio measurement ICAR, in an even closer analog to our past observations in stroke patients and animals after corticospinal tract lesions (Zaaimi et al., 2012; Choudhury et al., 2019).

### Clinical Implications

In older adults, a positive correlation between standardized grip strength and ICAR was seen, suggesting that those individuals who successfully maintain or enhance the RST are stronger. This correlation could arise from the effect of lifestyle. If a particular older subject regularly engaged in resistance training, we would expect this to increase muscle strength, and also to enhance RST output. Given the well-documented degeneration in the cortex and CST with aging, there may be no corresponding changes in cortical excitability, producing the elevated ICAR which we observed. However, it was of interest that the five older subjects with sarcopenia all had low ICAR ratios. This raises the intriguing possibility that the correlation between ICAR and normalized strength may reflect an underlying difference in the biology of age-related RST degeneration across our cohort, with a possible primary role in producing the sarcopenic state. Although this remains only a speculative possibility at the moment, it is a hypothesis worthy of further investigation. If correct, one prediction is that lowered RST output might predict those at risk of age-related muscle weakness and sarcopenia.

### Methodological Considerations

One limitation which must be acknowledged is that although the motivation for our study was to improve understanding of age-related muscle weakness, we were able to recruit only five sarcopenic patients in our community-based cohort. Still, this did have the advantage of placing changes in sarcopenia in the context of strength variation across the wider population.

It must also be noted that we measured grip strength as our primary measure of sarcopenia due to its widespread use and clinical validity in diagnosis, but iMEPs were elicited on biceps. However, grip strength correlates well with strength through the rest of the body including in the biceps muscle (Hyatt et al., 1990; Cruz-Jentoft et al., 2018). Eliciting iMEPs in the more distal muscles that are related to grip remains extremely challenging (Bawa et al., 2004). The current experimental design, therefore, seems a reasonable compromise.

Correlations between standardized grip strength and ICAR may be led by subjects with ICAR values >0.2; removal of these subjects from the dataset means the correlation was no longer significant in either age group. We reviewed this subgroup of subjects carefully, however, and found no reason to believe that there was any discrepancy in their task-performance, anthropometry, nor their behavior during the experiment. We have therefore included these data. Human strength is highly variable, particularly in older subjects (Dodds et al., 2014), and understanding the range of this variability is important to understand susceptibility to sarcopenia.

Earlier studies showed that the optimal site for iMEPs in biceps is often located anteromedial to that for cMEPs (Tazoe and Perez, 2014). Given the dynamic nature of the rowing task used to test iMEPs, it was impractical here to optimize the iMEP stimulation site, and instead, we used the cMEP hot spot. This may mean that the amplitude of iMEPs was underestimated, and likewise may increase iMEP latency and observed iMEP threshold due to increased distance of cortical tissue from the TMS focal point.

The resistance against which subjects rowed was fixed at 12 kg force, and it is highly possible that subjects were performing at different proportions of their maximal strength, and therefore different %MVCs. Higher levels of muscle activation are more likely to produce iMEPs (Ziemann et al., 1999). The fact that there was no relation between ICAR and raw strength nor LMM argues powerfully that our results are not simply driven by an artefactual effect of differences in contraction level as a percentage of each subject’s maximum.

Joint angle and muscle activity level (%MVC) were not measured during this experiment. However, this was a bilaterally symmetric movement, so that there cannot have been differences in muscle activity between the two sides. Both cMEPs and iMEPs were assessed following stimulation of the same motor cortex (left hemisphere); any changes in cortical excitability related to joint angle would therefore be the same for cMEPs and iMEPs. This might cause both measures to increase or decrease together but could not alter their ratio as measured by ICAR.

iMEP amplitude is partly dependent upon interhemispheric inhibition (Ferbert et al., 1992). It is therefore possible that the effects we have demonstrated are not due to the reticulospinal tract, but rather interhemispheric inhibition (IHI). To counter this, we have measured the peak iMEP amplitude, across varying TMS intensities, and measured the strongest iMEP response (where IHI is therefore lowest).

### Future Research

Finally, this work suggests a potential role of the RST for neurorehabilitation of individuals with sarcopenia. Techniques that stimulate the RST, including startling acoustic stimulus (Fernandez-Del-Olmo et al., 2014; Bartels et al., 2020) should be explored to measure the importance of neural adaptations on strength training in a cohort with age-related muscle weakness.

### Conclusion

Here, we have shown how age-related strength changes are related to the balance of descending motor drive between corticospinal and reticulospinal tract, as measured *via* non-invasive brain stimulation measure (ICAR).

## Data Availability Statement

The raw data supporting the conclusions of this article will be made available by the authors, without undue reservation.

## Ethics Statement

The studies involving human participants were reviewed and approved by Newcastle University Medical Faculty ethics committee. The patients/participants provided their written informed consent to participate in this study.

## Author Contributions

Both authors (SM and SB) are responsible for the experimental design, investigation, and preparation of this manuscript. All authors contributed to the article and approved the submitted version.

## Conflict of Interest

The authors declare that the research was conducted in the absence of any commercial or financial relationships that could be construed as a potential conflict of interest.
